# Perfluorooctanoic Acid (PFOA) Exposure in Early Life Increases Risk of Childhood Adiposity: A Meta-Analysis of Prospective Cohort Studies

**DOI:** 10.3390/ijerph15102070

**Published:** 2018-09-21

**Authors:** Pingping Liu, Fang Yang, Yongbo Wang, Zhanpeng Yuan

**Affiliations:** 1 Department of Preventive Medicine, School of Health Sciences, Wuhan University, Wuhan 430071, China; ppingliu@whu.edu.cn; 2 Department of Nursing, School of Health Sciences, Wuhan University, Wuhan 430071, China; yangfang95@163.com; 3 Department of Epidemiology and Biostatistics, School of Health Sciences, Wuhan University, Wuhan 430071, China; wangyb20172030@163.com; 4 Hubei Provincial Key Laboratory for Applied Toxicology, Wuhan 430065, China

**Keywords:** body mass index, childhood overweight, meta-analysis, perfluorooctanoic acid

## Abstract

Some articles have examined perfluorooctanoic acid (PFOA) exposure in early life in relation to risk of childhood adiposity. Nevertheless, the results from epidemiological studies exploring the associations remain inconsistent and contradictory. We thus conducted an analysis of data currently available to examine the association between PFOA exposure in early life and risk of childhood adiposity. The PubMed, EMBASE, and Web of Science databases were searched to identify studies that examined the impact of PFOA exposure in early life on childhood adiposity. A random-effects meta-analysis model was used to pool the statistical estimates. We identified ten prospective cohort studies comprising 6076 participants with PFOA exposure. The overall effect size (relative risk or odds ratio) for childhood overweight was 1.25 (95% confidence interval (CI): 1.04, 1.50; *I*^2^ = 40.5%). In addition, exposure to PFOA in early life increased the *z*-score of childhood body mass index (*β* = 0.10, 95% CI: 0.03, 0.17; *I*^2^ = 27.9%). Accordingly, exposure to PFOA in early life is associated with an increased risk for childhood adiposity. Further research is needed to verify these findings and to shed light on the molecular mechanism of PFOA in adiposity.

## 1. Introduction

The prevalence of obesity has increased worldwide over the past four decades, and is now a global health issue, with over 340 million children and adolescents aged 5–19 being overweight or obese in 2016 [1]. Overweight or obesity in children is more likely to persist into adulthood and further increases the likelihood of many health problems in later life, including obesity, cancer, diabetes, and cardiovascular disease [2,3]. Although obesity is known to be caused mainly by energy imbalance, growing evidence has suggested that exposure to other risk factors such as endocrine-disrupting chemicals may also play a role [4].

Perfluoroalkyl substances (PFASs) are a class of synthetic and highly stable compounds that are widely applied in protective coatings of food, textiles, furniture, and nonstick cookware [5,6,7]. They are known to act as endocrine disruptors [8], and early-life exposure is linked to the development of obesity [9]. Perfluorooctanoic acid (PFOA) is a typical member of the family of PFASs, which has unique and useful chemical properties including surface activity, thermal and acid resistance, and amphiphilic properties, and it has thus seen widespread use worldwide throughout the environment [10]. PFOA has been produced for over 50 years and has an estimated half-life of 3.5 years in human serum [11,12]. The main sources of human exposure to PFOA include diet, contaminated drinking water, food packaging and indoor house dust [13], as well as proximity to manufacturing [14]. Due to the persistence and bio-accumulative properties of PFOA and its adverse effects on humans, the manufacturing and emissions of PFOA have been phased out in many countries including the US [15], although PFOA has continued to be detected in the serum of most people in the US [16]. Moreover, in recent years, to meet the growing demand for commercial applications, PFOA manufacturing has increased in Asia [10], and the US has started the production of novel replacement chemistries that contain traditional perfluorinated carbon regions [17].

The literature exploring the relationship between exposure to PFOA and adiposity has increased dramatically over the past decade, with a particular focus on early-life exposure. However, the evidence is controversial. A prospective study of Faroese children 5 years of age (*n* = 444) observed a significant association between maternal serum concentrations of PFOA and overweight risk [9]. In contrast, some studies reported that exposure to PFOA in early life had no obvious role on childhood overweight risk [18,19]. In addition, no meta-analysis is currently available to quantitatively evaluate the relationship between exposure to PFOA in early life and childhood adiposity. Consequently, the main objective of this study was to evaluate the available epidemiological evidence linking childhood adiposity with early-life exposure to PFOA.

## 2. Materials and Methods

### 2.1. Literature Search

Keywords including PFOA, child, adiposity (full search terms in Table S1) were applied to three electronic literature databases, EMBASE, Web of Science, and PubMed, on 31 December 2017, and an updated on 19 May 2018. Searches were limited by English language, but publication date was uncontrolled. Additionally, the reference lists of conference abstracts, reviews and expert publications were also reviewed. The records were collected in Endnote X7 (Thomson Reuters, Toronto, ON, Canada) and filtered manually to eliminate duplicates.

### 2.2. Study Selection Criteria

Two reviewers (P.L. and F.Y.) independently conducted all studies by title or abstract, and then by a full-text screening. Any discrepancies among the two reviewers reached a consensus by discussion. Studies were included in the meta-analysis when they satisfied all of the following criteria: (1) the study was designed as a prospective cohort study; (2) offspring were exposed to PFOA in utero or in the first year of life (early-life exposure), and maternal serum or plasma concentrations served as proxies for early-life exposure; (3) the outcomes of interest were childhood overweight, obesity or body mass index (BMI); (4) the child’s weight and height were measured when the child was more than 2 years old, because BMI is a commonly used indicator of excess weight in children over 2 years old; and (5) provided risk estimates on the associations, such as relative risks (RRs), odds ratios (ORs), or beta regression coefficients (βs) with 95% confidence intervals (95% CIs), or provided sufficient data to allow adequate estimation of the RRs/ORs/βs and 95% CIs. Studies containing overlapping data or that did not report original data, such as reviews, comments, editorials, and meeting abstracts, were all excluded.

### 2.3. Data Extraction

Two reviewers (P.L. and F.Y.) extracted data from all of the included articles independently, and discrepancy was resolved by a third reviewer (Y.W.) for quality assurance and quality control. We collected details of selected articles, primarily including first author, study design, baseline time, location, outcome measurements, measurement timing, duration of follow-up, sample matrix and adjusted variables. When outcomes were measured at different follow-ups, we extracted the data for the longest follow-up time. The authors were contacted to request crucial information data not provided in the selected articles. If no response was received from the authors within one month, the data were excluded from analysis.

### 2.4. Quality Assessment

The quality evaluation of included studies was performed using the 9-star Newcastle–Ottawa scale, which includes 3 categories: selection (4 items, 0–4 points), comparability (1 item, 0–2 points), and outcome (3 items, 0–3 points) for cohort studies. Each category that meets the criteria can be awarded a star, except for the comparability category (which awards a maximum of 2 stars). A score of seven or more points was considered as high-quality study [20].

### 2.5. Statistical Analysis and Heterogeneity

The risk estimates on the association between early-life exposure to PFOA and childhood adiposity were pooled using meta-analysis. The *β* regression coefficients were used for childhood BMI *z*-score, and RRs and/or ORs were used for childhood overweight. When different *β*/RR/OR values for different percentiles, tertiles, or quartiles of PFOA instead of continuous trends were provided in included studies, the strongest association with the outcome (worst-case scenario) was selected [21]. The effect sizes were pooled using the inverse variance method for random-effects meta-analysis [22].

Heterogeneity was measured with the *I*^2^ statistic and was represented on a scale ranging from 0% to 100%. *I*^2^ values of 25%, 50%, and 75% were considered as low, medium, and high heterogeneity, respectively [23]. A random-effects model was used to summarize data to reduce the impact of heterogeneity between studies [22]. Subgroup analyses were carried out by effect size, measurement timing, geographical region, gender and with or without adjustment of maternal parity and birth weight to explore possible sources of heterogeneity. We also performed two sensitivity analyses. The first is to explore the impact of each individual study in our meta-analysis by excluding one study in turn and re-estimating the summary estimate. The second is to exclude the study of small sample sizes. We used Egger’s test to check the symmetry of the funnel plot to assess publication bias for childhood BMI, and *p* < 0.05 hints publication bias. Egger’s test detects funnel plot asymmetry by determining whether the intercept deviates significantly from zero in the regression equation based on the standard normal deviate and precision of each study included in the meta-analysis [24]. All statistical analyses were performed using STATA version 12.0 (StataCorp, College Station, TX, USA). A 2-sided *α* of 0.05 was considered statistically significant.

## 3. Results

### 3.1. Study Selection Flow

**Figure 1:**
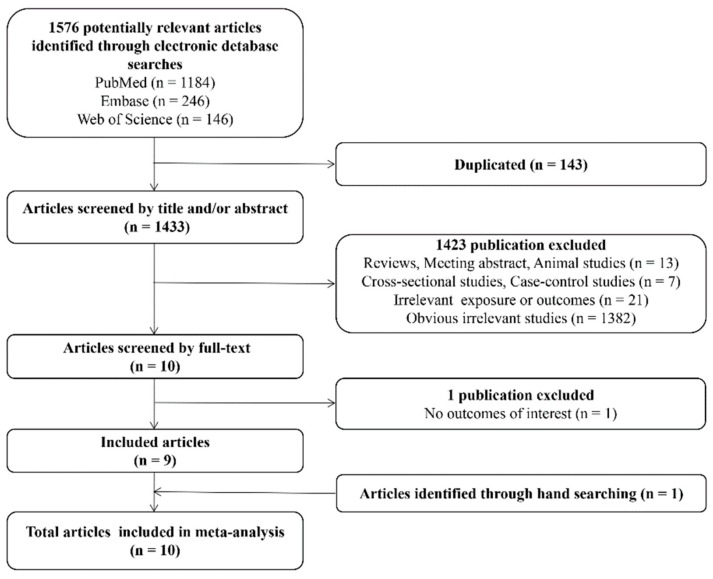
Flow diagram for search and selection of 10 included studies, for a meta-analysis of studies of exposure to perfluorooctanoic acid (PFOA) in early life and risk of childhood adiposity, published during 2012–2018.

Our search retrieved a total of 1576 articles, of which 1433 were retained after manual removal of 143 duplicates. After sifting titles and abstracts, we retained 10 records for full-text screening, resulting in 9 full-text peer-reviewed articles retained for data extraction; 1 study was further removed due to no outcomes of interest [25]. Specifically, the study by Wang et al. reported an average weight *z*-score across childhood but did not report the outcomes of interest such as childhood overweight, obesity or BMI. In addition, hand searching the reference lists of the 9 included articles identified 1 additional study, resulting in a total of 10 articles involving 6076 participants that were retained for quantitative synthesis in the meta-analysis [9,18,19,26,27,28,29,30,31,32]. The flow diagram of literature selection progression is shown in Figure 1.

### 3.2. Details of Included Studies

The characteristics of the 10 cohort studies are detailed in Table 1. The population size varied among the 10 included prospective cohort studies, ranging from 200 to 1086 participants. These studies covered the years from 1986 to 2011 and included populations located in nine regions.

As for outcomes assessment, different standards were used to calculate BMI *z*-score, including the International Obesity Task Force standards [30], the 2000 Centers for Disease Control and Prevention (CDC) Growth Charts [18,28], longitudinal reference value for the Swedish population from birth to 18 years old [27], or the World Health Organization (WHO) Growth Standards [9,19,29,31,32]. The definition of overweight or obesity was different among studies. Of the 10 included studies, 3 defined overweight as BMI *z*-score ≥ 85th percentile [9,19,29], 2 defined overweight or obesity as BMI *z*-score ≥ 85th percentile [28,32], 1 defined overweight or obesity as BMI ≥ 25 kg/m^2^ [26], 1 defined overweight for boys and girls as BMI ≥ 17.92 kg/m^2^ and 17.75 kg/m^2^, respectively [30], and 1 defined obesity as BMI ≥ 95th percentile, while overweight was defined as BMI ≥ 85th and < 95th percentile [18].

Among those 10 studies, nine analysed the influence of early-life exposure to PFOA on childhood BMI [9,18,19,27,28,29,30,31,32], four were on childhood overweight [9,18,19,29], one on childhood obesity [18], and four on childhood overweight or obesity [26,28,30,32]. The techniques for analysing PFOA were different. The quality of the 10 cohort studies is shown in Table S2.

### 3.3. Meta-Analysis of PFOA Exposure in Early Life in Relation to Childhood Overweight

**Figure 2:**
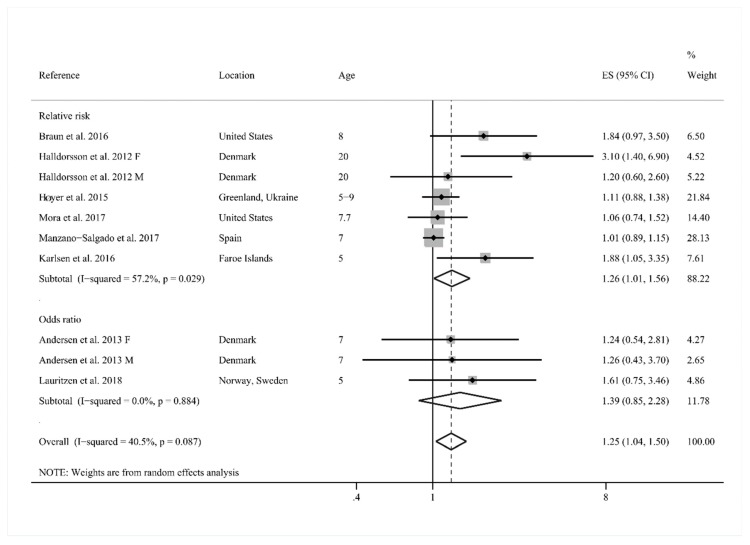
Forest plot of exposure to perfluorooctanoic acid (PFOA) in early life in relation to childhood overweight risk, stratified by effect size (RRs and ORs). ES, effect size; CI: confidence interval; F: females; M: males.

One of the 10 cohort studies reported overweight and obesity data, respectively. Overweight and obesity data were combined as overall overweight data because it is considered to be more effective (i.e., the overall overweight data has the strongest association with the PFOA exposure). As shown in Figure 2, exposure to PFOA in early life had a statistically significant association with childhood overweight risk (effect size = 1.25, 95% CI: 1.04, 1.50; *I*^2^ = 40.5%). When the studies stratified analysis by the effect size (relative risk vs. odds ratio), a significant correlation between early-life exposure to PFOA and childhood overweight risk was observed in group of relative risk (RR) (RR = 1.26, 95% CI: 1.01, 1.56; *I*^2^ = 57.2%), while the odds ratio (OR) group had a slightly higher assessment but no significant risk for childhood overweight (OR = 1.39, 95% CI: 0.85, 2.28; *I*^2^ = 0.0%). The pooled effect sizes for males and females were selected for all studies, with the exception of Halldorsson et al. [26] and Andersen et al. [30], where only overweight data of sex-specific childhood was reported and thus was plotted individually.

### 3.4. Meta-Analysis of PFOA Exposure in Early Life in Relation to Childhood BMI

**Figure 3:**
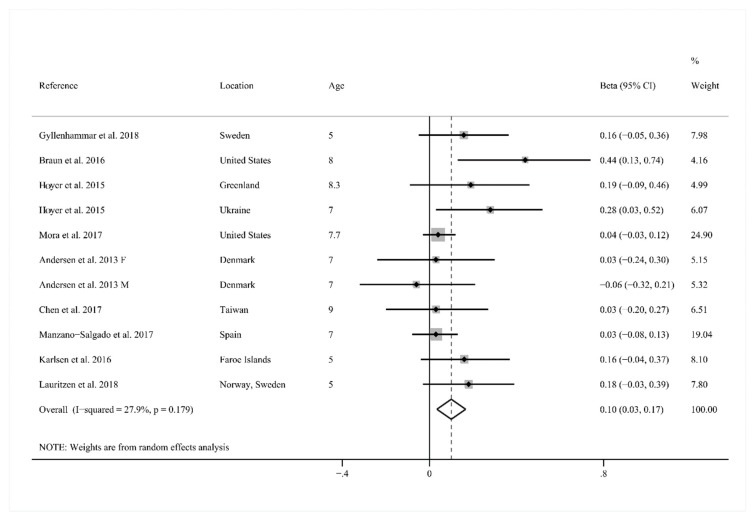
Forest plot of pooled *β* values for the association between exposure to perfluorooctanoic acid (PFOA) in early life and body mass index (BMI) *z*-score of childhood. CI: confidence interval; F: females; M: males.

Among those 10 studies, nine provided pooled *β* for childhood BMI *z*-score after early-life exposure to PFOA. One reported separate *β* for males and females, and thus was entered as two studies [30]. As shown in Figure 3, meta-analysis showed that PFOA exposure in early life could slightly increase the *z*-score of childhood BMI with a moderate heterogeneity (*β* = 0.10, 95% CI: 0.03, 0.17; *I*^2^ = 27.9%).

When the studies were stratified by measurement timing, both prenatal and postnatal exposure to PFOA indicated a small increase in the *z*-score of childhood BMI (prenatal exposure, *β* = 0.09, 95% CI: 0.02, 0.17; *I*^2^ = 36.3%, postnatal exposure, *β* = 0.16, 95% CI: 0.01, 0.30; *I*^2^ = 0.0%). A significant association between early-life exposure to PFOA and childhood BMI *z*-score was observed among the studies performed in Europe (*β* = 0.10, 95% CI: 0.02, 0.17; *I*^2^ = 8.4%), but not in Northern America (*β* = 0.19, 95% CI: −0.05, 0.42; *I*^2^ = 71.4%) and Asia (*β* = 0.03, 95% CI: −0.21, 0.27). Subgroup analysis of maternal parity revealed a significant positive association between early-life exposure to PFOA and childhood BMI *z*-score in the group adjusted by maternal parity (*β* = 0.13, 95% CI: 0.02, 0.24; *I*^2^ = 47.4%). Within the group by gender, PFOA exposure increased the *z*-score of childhood BMI in females (*β* = 0.06, 95% CI: −0.01, 0.13; *I*^2^ = 0), but not males (*β* = −0.01, 95% CI: −0.10, 0.08; *I*^2^ = 0), and neither achieved significant levels. In addition, the subgroup of birth weight was evaluated, and PFOA exposure could statistically significantly increase the *z*-score of childhood BMI in the group that was not adjusted by birth weight (*β* = 0.10, 95% CI: 0.03, 0.17; *I*^2^ = 31.9%) (Table 2).

### 3.5. Sensitivity Analysis

For childhood BMI, sensitivity analysis showed that the confidence intervals did not overlap the null in the eleven possible combinations (Figure S1). The number of participants was considered to result in imprecision for continuous variables when it was less than 400 [33]. Because the average number of participants in the included studies related to childhood BMI was approximately 490, it was suggested that imprecision was not serious. Moreover, when these studies with small sample sizes (9,27,28,30) were omitted in the sensitivity analysis, the conclusion did not change significantly (*β* = 0.07, 95% CI: 0.01, 0.14; *I*^2^ = 14.4%). In brief, the results of the sensitivity analysis did not alter the overall association of PFOA exposure and childhood adiposity.

### 3.6. Publication Bias

As shown in Figure S2, the funnel plot did not show obvious asymmetry, and Egger’s test did not reveal definite publication bias (P_Egger’s_ test = 0.054) (Table S3). However, the number of studies included was limited, which may lead to insufficient statistical testing, and thus, the possibility of publication bias cannot be ruled out.

## 4. Discussion

Currently, excessive food intake, inadequate exercise, endocrine-disrupting chemicals, and possible gene-environment interactions are major determinants of obesity. Evidence has shown that the critical window of exposure to endocrine-disrupting chemicals is either in the prenatal or neonatal period [34]. PFOA is suspected as an endocrine disruptor [35,36], and exposure to PFOA has at least three possible pathways that may affect the weight of offspring. First, the ovarian axis may be part of the mode of action for PFOA. This approach is supported by the observation that ovary removal in animals before puberty, combined with PFOA exposure, caused no additional increase in mid-life body weight [37]. Second, some experimental results have demonstrated that PFOA may activate peroxisome proliferator-activated receptor (PPAR) *α* or *γ*, which act as master lipid regulators to modify lipid homeostasis [38,39]. Third, PFOA may be involved in altering thyroid hormone homeostasis, which plays an important role in the regulation of energy metabolism. A meta-analysis of the association between perfluoroalkyl exposure and adult thyroid function showed a positive correlation between PFOA and total T3 and a significant negative correlation between PFOA concentration and total thyroxine, after omitting one outlier study [40]. However, the potential mechanism of action remains to be elucidated. Therefore, more studies are needed to shed light on the molecular mechanism of PFOA in obesity. 

To the best of our knowledge, this meta-analysis is the first study to evaluate the impact of early-life exposure to PFOA on childhood adiposity. A final total of ten prospective cohort studies were included. The results of our meta-analysis showed that early-life exposure to PFOA had statistically significant correlation with increased risk of childhood overweight and slightly increased the *z*-score of childhood BMI. Thus, the results of this meta-analysis demonstrated a positive overall association of PFOA exposure in early life with childhood adiposity.

CD-1 mice exposed to PFOA for 17 days (0.01 to 5 mg/kg body weight) during pregnancy showed that low-dose exposures (0.01–0.3 mg/kg/d) significantly increased body weight in mid-life of offspring [37]. The study also found that exposing a group of independent and age-matched adult-only female mice to similar doses of PFOA for same exposure time had no effect on body weight. These observations demonstrated that early life as a sensitive window of exposure for these low-dose effects of PFOA exposures on gain of body weight in female mice. This work on CD-1 mice preceded the work of human cohorts and showed the same trend as our present study.

Potential bias should be considered in the meta-analysis. Although the meta-analysis of early-life exposure to PFOA and child BMI showed moderate heterogeneity, we performed subgroup analyses to assess potential bias. Maternal parity is a known predictor of PFAA exposure [41], and being the firstborn may be associated with great adiposity in childhood [42]. It was necessary to adjust for maternal parity to explore the reliable relationship between PFOA exposure and childhood obesity because 4 out of the 11 included studies did not adjust for maternal parity [19,27,31,32]. A significant association was observed between early-life exposure to PFOA and the *z*-score of childhood BMI in the adjusted maternal parity group (*β* = 0.13, 95% CI: 0.02, 0.24), whereas the association was non-significant in the non-adjusted maternal parity group (*β* = 0.07, 95% CI: −0.01, 0.15). Therefore, this result suggested that maternal parity had a potential effect on childhood obesity, and the positive association between early-life exposure to PFOA and the *z*-score of childhood BMI might more truly express that association, further confirming the reliability and robustness of our results.

Quantification of PFOA in included studies was carried out in different matrices, 2 in postpartum maternal serum [9,27], 4 in prenatal maternal serum [26,28,29,32], 3 in prenatal maternal plasma [18,19,30] and 1 in cord blood [31]. Available evidence indicated that PFOA concentrations were highly correlated between these matrices [9,41,43]. Based on this concordance, we initially combined the studies in our meta-analysis, but analysis of the measurement timing was still conducted. The results from subgroup analysis of measurement timing revealed a similarly positive association between prenatal and postnatal exposure to PFOA and the *z*-score of childhood BMI, and both reached a statistically significant level (prenatal exposure, *β* = 0.09, 95% CI: 0.02, 0.17, postnatal exposure, *β* = 0.16, 95% CI: 0.01, 0.30). The results are broadly consistent with a previous study that demonstrated that PFOA concentrations were highly correlated between prenatal and neonatal periods [44].

This meta-analysis has several strengths. First, considering the uncontrollability of pollutant exposures and the ethical limitations on conducting controlled trials with pollutants in humans, cohort design is considered the most feasible method. One of the inclusion criteria of our meta-analysis was that the design type of the literature be a cohort study. Second, findings were consistent across multiple outcome types (i.e., childhood overweight and BMI), further supporting the robustness of the findings.

There are also several limitations in this meta-analysis. First, one limitation is the study design of the included articles. Sex-specific differences associated with weight gain have been observed for PFOA in some studies. As mentioned earlier, the animal study on CD-1 mice reported higher body weight in pospubertal female offspring after low-dose prenatal exposures to PFOA [37]. Human studies showed similar findings: Halldorsson et al. [26] observed a positive association between prenatal PFOA and overweight in young female adults, and Høyer et al. [29] indicated that higher prenatal PFOA concentrations were associated with an increased risk of overweight among girls in Greenland. In our meta-analysis, four of the nine studies related to childhood BMI were stratified for gender. Based on these four studies [18,19,30,31], we conducted a subgroup analysis of gender. The results of subgroup analysis showed that the *z*-score of childhood BMI in the female group was slightly elevated but not statistically significant (*β* = 0.06, 95% CI: −0.01, 0.13), while the male group showed the opposite trend (*β* = −0.01, 95% CI: −0.10, 0.08). This is probably due to the low number of related studies, the included studies were not sufficient to verify gender differences. Future study on this matter should address gender difference with appropriate design and larger sample size. Second, the study was based on the available data, and non-English articles were excluded, thereby making publication and selection biases likely inevitable. Third, it was reported that the distinctions among the OR and RR could be ignored when the outcome is rare [45]. Clearly, we did not transform RRs (ORs) into ORs (RRs) as the common effect size for assessment the childhood overweight, which might be a potential source of heterogeneity. Fourth, the criteria used to calculate BMI *z*-score varied across the studies, and the definition of overweight or obesity also varied, which may result in imprecise estimates of association between PFOA exposure and childhood adiposity. Fifth, birth weight is a known predictor of offspring BMI [42], but only one study included in our meta-analysis adjusted for birth weight. The results of subgroup analysis demonstrated that the adjusted group had a larger effect estimate, with wider CIs (adjusted group, *β* = 0.16, 95% CI: −0.05, 0.36, non-adjusted, *β* = 0.10, 95% CI: 0.03, 0.17). The association between PFOA exposure and childhood adiposity may be exaggerated due to inconsistent birth weight baseline. Sixth, most of the research comes from Europe (Norway, Denmark, Sweden, Spain, Ukraine, Faroe Islands) and North America (the United States, Greenland), with one study from Asia (Taiwan). There was a significant association between PFOA exposure and childhood adiposity in Europe (*β* = 0.10, 95% CI: 0.02, 0.17; *I*^2^ = 8.4%), but not in Northern America (*β* = 0.19, 95% CI: −0.05, 0.42; *I*^2^ = 71.4%) and Asia (*β* = 0.03, 95% CI: −0.21, 0.27), and these results suggested differences between ethnicities. Due to the lack of African data in our study, the findings may not be globally universal. Seventh, no consistent trend was observed after a preliminary examination of the dose-response trend for studies that met the dose-response meta-analysis conditions. We did not conduct dose-response meta-analysis, given the variability between different exposure and reference groups in different studies. In addition, to reduce the impact of confounding factors, we extracted data reported from adjusted models, which means that our results may be susceptible to over-adjustment.

With the increasing prevalence of childhood obesity in recent years, effective treatments have not been found, and prevention remains crucial. Exploring the association between PFOA and childhood obesity may help researchers further understand the consequences of early-life exposure to PFOA on offspring obesity and may also provide effective prevention or treatment measures for childhood adiposity, such as reducing risk of exposure to PFOA through early education. Thus, an improved understanding of the association between early-life exposure to PFOA and child adiposity has important implications for public health.

## 5. Conclusions

In summary, limited to prospective cohort studies, our meta-analysis indicated a statistically significant association between early-life exposure to PFOA and childhood adiposity. However, the results may be affected by other factors, such as we cannot rule out the possibility that these associations were caused by accumulation or synergy of multiple chemical exposures or by potential residual confounding factors. Future research with prudent design is warranted to verify these findings.

## Figures and Tables

**Table 1:** Characteristics of prospective studies on the association between exposure to perfluorooctanoic acid (PFOA) in early life and child adiposity.

Cohort	Reference	Location (Baseline Time)	Study Design	*N*	Gender	Age	Analysis of PFOA	Measurement Timing	Sample Matrix	Confounders	Outcomes of Interest
POPUP	Gyllenhammar et al. 2018 [27]	Uppsala County (Sweden) (1996–2011)	Prospective cohort	200	FM	5	UPLC-MS/MS	3 weeks postpartum	Maternal serum	Sampling year, maternal age, pre-pregnancy BMI, gestational weight gain, maternal weight loss after delivery, education, breastfeeding	BMI standard deviation scores
HOME Study	Braun et al. 2016 [28]	Cincinnati, Ohio (USA) (2003–2006)	Prospective cohort	204	FM	8	HPLC-ID/MS/MS	16, 26 weeks or birth	Maternal serum	Maternal age, race, education, income, parity, employment, marital status, depressive symptoms, BMI at 16 weeks gestation, fruit consumption, vegetable consumption, fish consumption, prenatal vitamin use, maternal serum cotinine	Overweight or obesity; BMI *z*-score
Aarhus Birth Cohort	Halldorsson et al. 2012 [26]	Denmark (1988–1989)	Prospective cohort	665	F/M	20	cLC-MS/MS	30 weeks	Maternal serum	Maternal age, education, pre-pregnancy BMI, smoking, parity, birth weight, age	Overweight or obesity
INUENDO	Høyer et al. 2015 [29]	Greenland Ukraine (2002–2004)	Prospective cohort	531 491	F/M/FM	8.3 7	LC-MS/MS	25 weeks 23 weeks	Maternal serum	Maternal age, pre-pregnancy BMI, smoking, education, parity	Overweight; BMI *z*-score
Project Viva	Mora et al. 2017 [18]	Massachusetts (USA) (1999–2002)	Prospective pre-birth cohort	876	F/M/FM	7.7	HPLC-ID/MS/MS	9.6 weeks	Maternal plasma	Maternal age, race, education, parity, pre-pregnancy BMI, gestational age, income, sex, age	Overweight; Obesity; BMI *z*-score
Danish National Birth Cohort	Andersen et al. 2013 [30]	Denmark (1996–2002)	Prospective cohort	811	F/M	7	HPLC-MS/MS	8 weeks	Maternal plasma	Age, maternal age, parity, pre-pregnancy BMI, smoking, socioeconomic status, gestational age	Overweigh or obesity; BMI *z*-score
TBPS	Chen et al. 2017 [31]	Taiwan (China) (2004–2005)	Prospective cohort	429	F/M/FM	9	UHPLC-MS/MS ^1^	At birth	Cord blood	Maternal age, pre-pregnancy BMI, education, log (Ln) transformed cord blood cotinine levels, sex, preterm birth	BMI *z*-score
INMA	Manzano-Salgado et al. 2017 [19]	Gipuzkoa Sabadell Valencia (Spain) (2003–2008)	Prospective cohort	280 382 424	F/M/FM	7	cLC-MS/MS	1st trimester	Maternal plasma	Maternal region of residence, birth country, previous breastfeeding, maternal age, pre-pregnancy BMI, age, sex	BMI *z*-score, Overweight
Faroe Islands Cohort	Karlsen et al. 2016 [9]	Faroe Islands (2007–2009)	Prospective cohort	371	FM	5	LC-MS	2 weeks postpartum	Maternal serum	Maternal region of residence, maternal age, pre-pregnancy BMI, gestational weight gain, parity, smoking, fish consumption, type of delivery, sex, birth weight	BMI *z*-score; Overweight
SGA Study	Lauritzen et al. 2018 [32]	Norway Sweden (1986–1988)	Prospective cohort	254 158	FM	5	UHPLC-MS/MS ^2^	2nd trimester	Maternal serum	Maternal age, education, smoking, pre- pregnancy BMI, weight gain at 17 weeks, inter-pregnancy interval, previous breastfeeding, maternal region of residence	BMI *z*-score; Overweight or obesity

BMI, body mass index; cLC-MS/MS: column-switching liquid chromatography coupled with tandem mass spectrometry; FM, females and males combined, F/M, females and males stratified; HPLC-MS/MS: high-performance liquid chromatography tandem mass spectrometry; HPLC-ID/MS/MS: high performance liquid chromatography-isotope dilution tandem mass spectrometry; INMA, INfanciay Medio Ambiente, Environment and Childhood birth cohort; LC-MS: liquid chromatography with mass spectrometry; LC-MS/MS: liquid chromatography–tandem mass spectrometry; POPUP, Persistent Organic Pollutants in Uppsala Primiparas; SGA, Scandinavian Successive Small-for-Gestational Age births; TBPS: Taiwan Birth Panel Study; UHPLC-MS/MS ^1^: ultra-high-performance liquid chromatography/tandem mass spectrometry; UPLC-MS/MS: ultra performance liquid chromatography-tandem mass spectrometry; UHPLC-MS/MS ^2^: ultrahigh pressure liquid chromatography triple-quadruple mass-spectrometry.

**Table 2:** Meta-Analysis of the Association between Exposure to Perfluorooctanoic Acid (PFOA) in Early Life and Childhood Body Mass Index (BMI).

Subgroup	Reference (Participants)	*β*	95% CI	*I*^2^ (%)	*p*-Value for Heterogeneity
Total studies	11 (6076)	0.10	0.03, 0.17	27.9	0.179
Prenatal exposure	9 (5505)	0.09	0.02, 0.17	36.3	0.128
Postnatal exposure	2 (571)	0.16	0.01, 0.30	0	0.989
Europe	7 (3545)	0.10	0.02, 0.17	8.4	0.364
Northern America	3(2102)	0.19	−0.05, 0.42	71.4	0.030
Asia	1 (429)	0.03	−0.21, 0.27	NA	NA
Adjusted by maternal parity Yes	7 (3949)	0.13	0.02, 0.24	47.4	0.077
Adjusted by maternal parity No	4 (2127)	0.07	−0.01, 0.15	0	0.491
Adjust birth weight Yes	1 (371)	0.16	−0.05, 0.36	NA	NA
Adjust birth weight NO	10 (5705)	0.10	0.03, 0.17	31.9	0.153
Female	4(1549)	0.06	−0.01, 0.13	0	0.573
Male	4(1628)	−0.01	−0.10, 0.08	0	0.712

CI: confidence interval; NA: not available.

## Supplementary Materials

The following are available online at http://www.mdpi.com/1660-4601/15/10/2070/s1: Figure S1: Results of the sensitivity analysis on the effects of early-life exposure to perfluorooctanoic acid (PFOA) on childhood body mass index (BMI) *z*-score; Figure S2: Funnel plot for studies of publication bias on the association between early-life exposure to perfluorooctanoic acid (PFOA) and childhood body mass index (BMI) *z*-score; Table S1: Search Terms Used in Systematic Literature Search; Table S2: Quality Assessments of the Prospective Cohort Studies Relating Perfluorooctanoic Acid (PFOA) and Child Adiposity; Table S3: Results of Egger’s Test in the Meta-Analysis.
