# Supplementary material for: Perfluorooctanoic Acid (PFOA) Exposure in Early Life Increases Risk of Childhood Adiposity: A Meta-Analysis of Prospective Cohort Studies
Source: Int J Environ Res Public Health. 2018 Sep 21;15(10):2070. doi: 10.3390/ijerph15102070 (PMC6209901; doi:10.3390/ijerph15102070)
Supplement: Supplementary file 1 [file ijerph-15-02070-s001.pdf]

Supplementary Materials

Perfluorooctanoic Acid (PFOA) Exposure in Early Life Increases Risk of Childhood Adiposity: A Meta-Analysis of Prospective Cohort Studies

Pingping Liu [1], Fang Yang [2], Yongbo Wang [3] and Zhanpeng Yuan [1,4,*]

1 Department of Preventive Medicine, School of Health Sciences, Wuhan University, Wuhan 430071, China; ppingliu@whu.edu.cn
2 Department of Nursing, School of Health Sciences, Wuhan University, Wuhan 430071, China; yangfang95@163.com
3 Department of Epidemiology and Biostatistics, School of Health Sciences, Wuhan University, Wuhan 430071, China; wangyb20172030@163.com
4 Hubei Provincial Key Laboratory for Applied Toxicology, Wuhan 430065, China
* Correspondence: zpyuan@whu.edu.cn; Tel.: +86-027-68759291

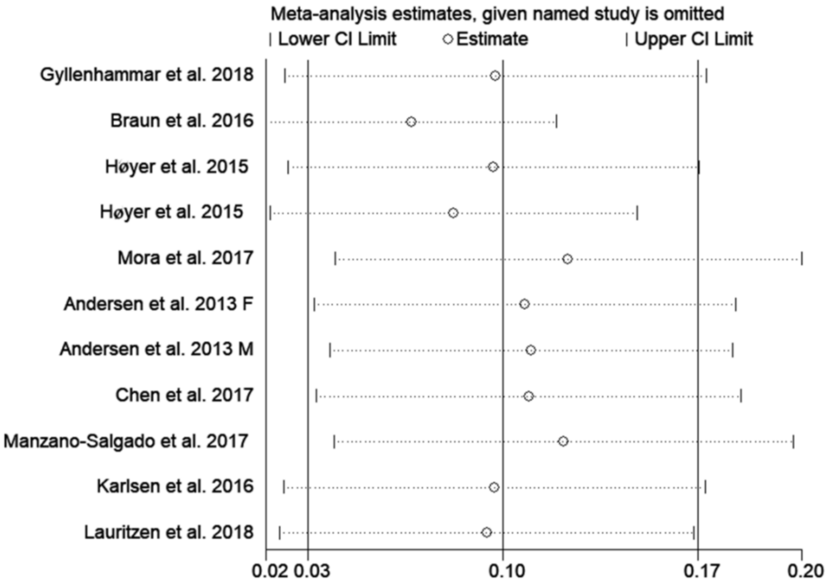

Figure S1. Results of the sensitivity analysis on the effects of early-life exposure to perfluorooctanoic acid (PFOA) on childhood body mass index (BMI) *z*-score.

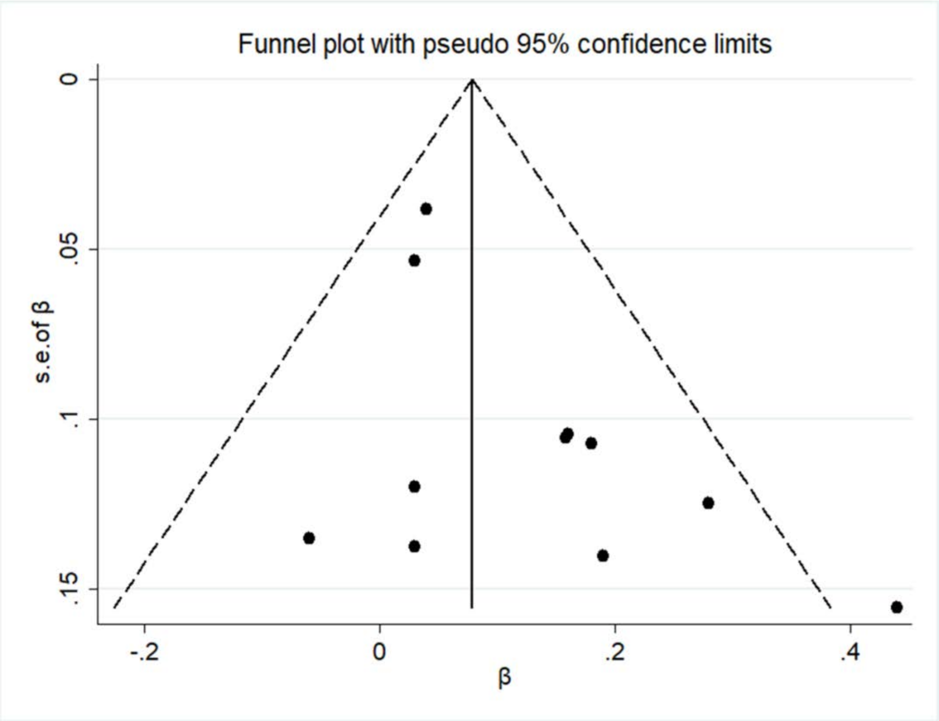

Figure S2. Funnel plot for studies of publication bias on the association between early-life exposure to perfluorooctanoic acid (PFOA) and childhood body mass index (BMI) *z*-score.

Table S1. Search Terms Used in the Systematic Literature Search.

Search	PubMed
#1 The exposure	335-67-1 [rn] OR perfluorooctanoic acid [nm] OR (perfluorooctanoic acid [tiab] OR perfluorooctanoic acids [tiab]) OR (perfluoroctanoic acid [tiab] OR perfluoroctanoic acids [tiab]) OR (perfluoro-n-octanoic acid [tiab] OR perfluoro-n-octanoic acids [tiab]) OR (pentadecafluorooctanoic acid [tiab] OR pentadecafluorooctanoic acids [tiab]) OR APFO [tiab] OR (perfluorinated [tiab] AND octanoic acid [tiab]) OR (perfluorinated [tiab] AND octanoic acids [tiab]) OR (perfluorooctanoate [tiab] OR perfluorooctanoates [tiab]) OR perfluorooctanoyl chloride [tiab] OR PFOA [tiab] OR (fluorinated telomer alcohol [tiab] OR fluorinated telomer alcohols [tiab]) OR (fluoro-telomer alcohol [tiab] OR fluoro-telomer alcohols [tiab]) OR (fluorocarbon emulsion [tiab] OR fluorocarbon emulsions [tiab]) OR (perfluorocarbon [tiab] OR perfluorocarbons [tiab]) OR (fluorocarbon polymer [tiab] OR fluorocarbon polymers [tiab]) OR (fluorinated polymer [tiab] OR fluorinated polymers [tiab]) OR octanoic acids [mh] OR (octanoic acid [tiab] OR octanoic acids [tiab]) OR caprylates [mh] OR (caprylate [tiab] OR caprylates [tiab]) OR (polyfluoroalkyl [tiab] OR polyfluoroalkyls [tiab] OR polyfluoroalkylated [tiab]) OR PFAA [tiab] OR (perfluoroalkyl chemical [tiab] OR perfluoroalkyl chemicals [tiab]) OR (c8 [tiab] AND perfluorinated [tiab]) OR (fluoropolymer [tiab] OR fluoropolymers [tiab] OR fluoropolymeric [tiab]) OR (fluorosurfactant [tiab] OR fluorosurfactants [tiab]) OR (perfluorochemical [tiab] OR perfluorochemicals [tiab]) OR PFCs [tiab] OR (perfluoroalkyl carboxylate [tiab] OR perfluoroalkyl carboxylates [tiab]) OR (perfluorocarboxylate [tiab] OR perfluorocarboxylates [tiab]) OR PFCA [tiab] OR (perfluorinated carboxylic acid [tiab] OR perfluorinated carboxylic acids [tiab]) OR FC 143 [tiab] OR (pentadecafluorooctanoate [tiab] OR pentadecafluorooctanoates [tiab])
#2 The health problem	adiposity [tiab] OR obesity [tiab] OR obese [tiab] OR overweight [tiab] OR body mass index [tiab] OR BMI [tiab] OR "quetelet index" [tiab] OR weight gain [tiab] OR body weight [tiab]
#3 Human study	child [tiab] OR children [tiab] OR childhood [tiab] OR boy* [tiab] OR girl* [tiab] OR infancy [tiab] OR infants [tiab] OR infant [tiab]
#4 Total	#1 AND #2 AND #3
	EMBASE
#1 The exposure	'perfluorooctanoic acid' OR 'perfluoro-n-octanoic acid' OR 'pentadecafluorooctanoic acid' OR apfo OR 'perfluorinated octanoic acid' OR perfluorooctanoate OR 'perfluorooctanoyl chloride' OR pfoa OR 'fluorinated telomer alcohol' OR 'fluorinated telomer alcohols' OR 'fluoro telomer alcohol' OR 'fluoro telomer alcohols' OR 'fluorocarbon emulsion' OR perfluorocarbon* OR 'fluorocarbon polymer' OR 'fluorocarbon polymers' OR 'fluorinated polymer' OR 'fluorinated polymers' OR polyfluoroalkyl* OR pfaa OR 'perfluoroalkyl chemical' OR 'perfluoroalkyl chemicals' OR c8 OR perfluorochemical* OR pfcs OR 'perfluoroalkyl carboxylate' OR perfluorocarboxylate OR pfca OR 'perfluorinated carboxylic acid' OR pentadecafluorooctanoate
#2 The health problem	adiposity OR obesity OR obese OR overweight OR 'body mass index' OR BMI OR 'quetelet index' OR 'weight gain' OR 'body weight'

Table S1. *Cont.*

#3 Human study	child OR children OR childhood OR boy * OR girl * OR infancy OR infants OR infant
#4 Total	#1 AND #2 AND #3
	Web of Science
#1 The exposure	TS=(perfluorooctanoic acid * OR perfluoro-n-octanoic acid * OR pentadecafluorooctanoic acid * OR APFO OR perfluorinated and octanoic acid* OR perfluorooctanoate * OR perfluorooctanoyl chloride OR PFOA OR fluorinated telomer alcohol * OR fluoro-telomer alcohol * OR fluorocarbon emulsion * OR perfluorocarbon * OR fluorocarbon polymer * OR fluorinated polymer * OR octanoic acid * OR caprylate * OR polyfluoroalkyl * OR PFAA OR perfluoroalkyl chemical * OR c8 and perfluorinated OR fluoropolymer * OR fluorosurfactant * OR perfluorochemical * OR PFCs OR perfluoroalkyl carboxylate * OR perfluorocarboxylate * OR PFCA OR perfluorinated carboxylic acid * OR pentadecafluorooctanoate *)
#2 The health problem	TS=(adiposity OR obesity OR obese OR overweight OR body mass index OR BMI OR quetelet index OR weight gain OR body weight)
#3 Human study	TS=(child OR children OR childhood OR boy * OR girl * OR infancy OR infants OR infant)
#4 Total	#1 AND #2 AND #3 NOT SO=polymer *

Table S2. Quality Assessments of the Prospective Cohort Studies Relating Perfluorooctanoic Acid (PFOA) and Child Adiposity.

Study	Selection				Comparability	Outcome			Overall quality score
	1	2	3	4	5	6	7	8	
Gyllenhammar et al. 2018	★	★	★	★	★	★	★	-	7
Braun et al. 2016	-	★	★	★	★★	★	★	-	7
Halldorsson et al. 2012	★	★	★	★	★★	-	★	★	8
Høyer et al. 2015	★	★	★	★	★★	-	★	★	8
Mora et al. 2017	★	★	★	★	★★	★	★	-	8
Andersen et al. 2013	★	★	★	★	★★	-	★	-	7
Chen et al. 2017	★	★	★	★	★	★	★	★	8
Manzano-Salgado et al. 2017	★	★	★	★	★	★	★	-	7
Karlsen et al. 2016	-	★	★	★	★★	★	★	-	7
Lauritzen et al. 2018	★	★	★	★	★	★	★	-	7

The study quality was assessed according to the Newcastle Ottawa Quality assessment scale for cohort studies. A maximum of 9 points to each study can be given in this scale: 4 for selection, 2 for comparability, and 3 for outcomes. 1, representativeness of the exposed cohort; 2, non-exposed cohort drawn from the same community; 3, ascertainment of exposure; 4, outcome of interest not present at start of study; 5, cohorts comparable on basis of the design or analysis (one star select the most important factor, two stars study controls for any additional factor); 6, assessment of outcome; 7, follow-up long enough for outcomes to occur; and 8, adequacy of follow up of cohorts.

Table S3. Results of Egger's Test in the Meta-Analysis.

Std_Eff	Coef.	Std. Err.	*t*	*P*>|t|	95% Conf. Interval	
**Slope**	−0.0218367	0.0517443	−0.42	0.683	−0.1388903	0.0952169
**Bias**	1.39341	0.6303908	2.21	0.054	−0.0326325	2.819454
